# Isolation and characterization of phages ΦZC2 and ΦZC3 against carbapenem-resistant *Acinetobacter baumannii*, and efficacy of ΦZC3 on A549 cells

**DOI:** 10.1186/s12985-025-02885-6

**Published:** 2025-07-30

**Authors:** Kareem Essam, Azza G. Kamel, Bishoy Maher Zaki, Mohamed Elhadidy, Amal Ahmed Abdel Aziz, Aysam Fayed, Tamer Roshdy, Ayman El-Shibiny

**Affiliations:** 1 Center for Microbiology and Phage Therapy, Zewail City of Science and Technology, Giza, 12578 Egypt; 2 Bioscience Research Laboratories Department, MARC for Medical Services and Scientific Research, Giza, Egypt; 3 Biomedical Sciences Program, University of Science and Technology, Zewail City of Science and Technology, October Gardens, 6th of October City, Giza, 12578 Egypt; 4 Department of Bacteriology, Mycology and Immunology, Faculty of Veterinary Medicine, Mansoura University, Mansoura, Egypt; 5 Genetic Engineering and Biotechnology Research Institute, University of Sadat City, El Sadat City, Egypt; 6 Medical Laboratory Techniques Department, College of Health and Medical Technique, Al-Mustaqbal University, Babylon, 51001 Iraq; 7 Faculty of Environmental Agricultural Sciences, Arish University, Arish, 45511 Egypt

**Keywords:** *Acinetobacter baumannii*, Phage therapy, Multidrug-resistant bacteria, ESKAPEE, Antibiotic resistance

## Abstract

**Background:**

*Acinetobacter baumannii* is an opportunistic pathogen and a major causative agent of hospital-acquired infections. This pathogen can acquire various antibiotic resistance genes, including those conferring resistance to last-resort antibiotics such as carbapenems. MDR *A. baumannii* is known to cause several infections, including pneumonia and urinary tract infections. Consequently, there is an urgent need to explore alternative therapies, and bacteriophage (phage) has emerged as a promising therapeutic approach for combating multidrug-resistant (MDR) infections.

**Materials and methods:**

This study investigates the therapeutic potential of specific bacteriophages against MDR, particularly carbapenem-resistant *A. baumannii*, and evaluates lytic activity against 41 clinical isolates of MDR *A. baumannii*. The phages morphotypes were identified by transmission electron microscope. The stability of these phages was assessed under different conditions, including pH (2, 3, 4, 7, and 10–12), temperature (-80, -20, 4, 37, 50, 60, 70, and 80 ^o^C), UV exposure (15, 30, 45, 60. 75, 90). Their antibacterial activity was also evaluated using a time-killing assay. Bacteriophage Insensitive Mutants (BIM) was assessed by MOI of 100. Genomic characterization was performed to predict protein-coding genes, life cycle, and suitability for therapeutic applications. Additionally, the safety and therapeutic efficacy of the phage were assessed using a cell viability MTT assay on adenocarcinomic human alveolar basal epithelial (A549) cells to evaluate the ability to rescue the lung cells from infection.

**Results:**

Two phages, vB_AbaP_ZC2 (ΦZC2) and vB_AbaM_ZC3 (ΦZC3), were isolated from hospital wastewater in Egypt. The phages demonstrated lytic activity against 24.3% (*n* = 10) and 31.7% (*n* = 13) of the isolates, respectively. Phage ΦZC2 demonstrated high EOP values (0.75–1) against AB23 and AB26, moderate activity on AB34 and AB35 (EOP = 0.19), and low or no activity on AB10, AB24, and AB31. Similarly, phage ΦZC3 exhibited high EOP on AB24 (EOP = 1), moderate levels on AB12, AB29, and AB38, while showing low or no efficacy against the remaining tested isolates. The morphotypes of ΦZC2 and ΦZC3 are podovirus and myovirus, respectively. The two phages were amplified using a bioreactor and reached titers of approximately 10¹⁰ PFU/ml in 2 L.ΦZC2 was stable at a pH range from 3 to 12 approximately 10^8^ PFU/ml, while ΦZC3 was stable at a pH range from 3 to 11 approximately 10^9^ PFU/ml compared to pH 7. ΦZC2 was stable at -80, 37, and 50 °C approximately 10^8^ PFU/ml, while ΦZC3 was stable at -80, 37,50, 60, and 70 °C with approximately 10^9^ PFU/ml compared to 4 °C. Additionally, the ΦZC2 phage exhibited stability at 90 min, while ΦZC3 phage exhibited stability at 75 min of exposure to UV light. The optimum MOI at which the ΦZC2 and ΦZC3 significantly reduced bacterial growth 0.1 and 0.01, respectively. The BIM frequency was higher for phage ΦZC3 compared to ΦZC2, indicating a slightly greater emergence of phage-resistant mutants with ΦZC3. Whole genome sequencing and annotation did not identify markers for lysogeny or antibiotic resistance. Phylogenetic analysis classified ΦZC2 and ΦZC3 within the genera of *Obolenskvirus* and *Friunavirus*, respectively. ΦZC3 was selected for its broad host range to be evaluated for rescuing A549 cells from MDR *A. baumannii* infection. ΦZC3 phage was not cytotoxic to A549 cells and rescued lung cells cocultured, reducing the concentration of bacteria by approximately 5 logs with different MOIs, after 6 h of incubation.

**Conclusion:**

In this study, the two lytic phages have antibacterial activity against MDR *A. baumannii*. particularly, ΦZC3 can be a potential therapy for pulmonary infections.

## Introduction

*Acinetobacter baumannii* (*A. baumannii)* is known for being virulent, as it was originally found in the environment and has a high ability to acquire resistance genes [1]. Over the last two decades, *A. baumannii* has tolerated the hospital environment and become one of the main multidrug-resistant (MDR) pathogens causing nosocomial infections [1]including pneumonia and urinary tract. *A. baumannii* is a Gram-negative bacterium that has emerged and globally spread since the Iraq War in 2003 [2, 3]. More data have indicated that drug-resistant isolates of MDR *A. baumannii* are more common and occur in countries that are both developed and developing [4]as misuse and overuse of antibiotics during the COVID-19 epidemic has recently become a significant concern for the risk of an increase in antimicrobial resistance (AMR) [5]. Reflecting this concern, Carbapenem-resistant isolates of *A. baumannii* have been repeatedly grouped within critical pathogens by the World Health Organization (WHO) in its reports for the Bacterial Priority Pathogens, and listed MDR *A. baumannii* as a significant priority of the ESKAPEE *(Enterococcus faecium*,* Staphylococcus aureus*,* Klebsiella pneumoniae*,* A. baumannii*,* Pseudomonas aeruginosa*,* Enterobacter species*, and *E. coli)* [6, 7].

MDR *A. baumannii* is becoming more challenging to control and treat, resulting in increased morbidity and longer durations of hospitalization and mortality, as it is highly capable of forming biofilms, which are bacterial communities embedded in a matrix of extracellular polysaccharides that the bacteria produce on their own [8, 9]. These biofilms allow the bacteria to attach to hospital surfaces and medical equipment. The most prevalent way nosocomial MDR *A. baumannii* infections spread is through breathing with a ventilator. Ventilator-associated pneumonia (VAP) has a mortality rate that ranges from 40–70% [10].

MDR *A. baumannii* acquires resistance in various ways [11] one of which is the enzymatic degradation of antibiotics [12]enzymes that degrade antibiotics. Included in these, β-lactamases are classified into four classes: Class A enzymes hydrolyze penicillin and include carbapenemases [13]Class B metallo-β-lactamases hydrolyze penicillins, cephalosporins, and carbapenems [14]Class C hydrolyzes cephalosporins [15]and Class D (OXA-type) oxacillinase enzymes hydrolyze carbapenems. Carbapenem-resistant *A. baumannii* (CRAB) is well-known for producing these enzymes due to the presence of these genes: OXA-23, OXA-24/40, and OXA-58 [16]. The CDC has reported that infections caused by CRAB do not respond to commonly used antibiotics, and some CRAB isolates are resistant to all available antibiotics [17] — leaving limited therapeutic options, primarily colistin. However, colistin-resistant isolates of MDR *A. baumannii* are increasing [18]. Considering the increase in infections and significant drug resistance, it is essential to develop new therapies for MDR *A. baumannii* infections, particularly carbapenem-resistant isolates [19] such as using bacteriophage therapy.

Bacteriophages (phages for short) are prokaryotic viruses that specifically target and infect their bacterial hosts [20]. Phages mostly follow either a lytic or lysogenic life cycle, and accordingly, phages can be classified into virulent or temperate [21]. In the lytic cycle, phage DNA is transcribed, replicated, and packaged into capsids forming a progeny of assembled virions. Some of the phage genes encode proteins like endolysins and holins that degrade the bacterial cell and release mature virions into the environment [22].Virulent phages can strictly undergo lytic life cycle, while temperate phages can do lytic cycle, they favor lysogeny in which they integrate their DNA into the host bacterial DNA. Temperate phages have specific lysogeny-related genes that regulate the switch between the life cycles, for example, transcription repressor-encoding genes that prevent the transcription of the lysis cassette, favoring lysogeny [21]. Therefore, virulent phages are preferred for therapeutic applications to ensure bacterial host lysis.

Since the pre-antibiotic era, virulent phages have been used to treat bacterial infections in humans and animals [23]. Phages are naturally ubiquitous in the environment and recognized as the earth’s most abundant biological agent [24]. Using virulent phages, which are extremely specific to their targets, is a promising approach to control the growth of MDR *A. baumannii* isolates [25]. In recent years, phage therapy has undergone a revival as a potentially effective strategy for combating antimicrobial-resistant pathogens and has shown therapeutic efficacy against MDR *A. baumannii* [26, 27]. However, it still faces several challenges, including its limited host range, the absence of established regulatory guidelines, and insufficient pharmacokinetic data [28].

Multiple studies have examined the antibacterial activity of phages using cell lines, including A549 cells. The A549 human alveolar basal epithelial cell line is useful in vitro model [29].These models provide valuable insights into the interaction between phages and bacterial pathogens, allowing researchers to assess the efficacy of phage therapy under controlled conditions. Each cell line offers distinct advantages for studying various aspects of phage-host interactions and their potential applications in antibacterial treatments [27, 30–32]. The effectiveness of phage was investigated using a human A549 alveolar epithelial cell culture. Experimental protocols included the treatment of A549 cells with various phage concentrations, followed by assessments of cytotoxicity and lytic activity in cell morphology [33]. The main objective of this study is to isolate and characterize phages that are effective against MDR *A. baumannii* and evaluate their antibacterial efficacy against MDR *A. baumannii* using an A549 cell line.

## Materials and methods

### Bacterial isolates and growth conditions

Forty-one MDR *A. baumannii* clinical isolates were obtained from sputum and respiratory secretions of hospitalized patients in Egypt. These isolates were provided by Mabaret Elasafra clinical microbiology laboratory serving multiple hospitals in Alexandria, Egypt, between August 2020 and April 2021, where they had been pre-identified and characterized, with species-level identification conducted using the VITEK2 Compact system (bioMérieux, Marcy-l’Étoile, France). The presence of carbapenemase-encoding genes was further confirmed by PCR with specific primers [34].

The collected bacterial isolates were sub-cultured on MacConkey agar (Oxoid, England), and they were preserved in tryptic soy broth (TSB; Merck, USA) supplemented with 25% glycerol for long-term storage at -80 °C.

### Bacteriophage isolation and characterization

#### Isolation and purification of bacteriophages

The protocol described by Clokie and Kropinski (2009) was used for phage isolation with minor modifications [35]. The wastewater samples were centrifuged in a 50 ml centrifuge tube at 5,000 × g for 20 min at 4 °C. For enrichment, 5 ml supernatant of wastewater was transferred to a sterile centrifuge tube with an equal volume of the TSB 2X in a sterile flask with 100 µl of mixed culture (AB20, AB21, AB22, AB23, AB24, AB25, AB26, AB27, AB28, and AB29) of *A. baumannii* isolates for overnight in a shaking incubator at 37 °C. The enrichment was transferred to 50 ml centrifuge tube and centrifuged at 5,000 ×g for 20 min at 4 °C. The supernatant was filtered by a 0.45 μm syringe membrane filter (Membrane Solution, USA). Double agar overlay plaque assay was used to evaluate bacteriophage activity; an overnight bacterial culture was combined with molten soft nutrient agar with a concentration of 0.75% agar and poured onto a 1.5% agar plate. 10 µl of supernatant was dropped onto the plate and incubated overnight at 37°C [36].

The formed plaques were purified by picking the single plaques from the plate with a sterile pipette tip and transferred to sterile SM buffer (100 mM NaCl, 8 mM MgSO_4_ • 7H_2_O, 50 mM Tris-Cl; pH 7.5) to elute the phage overnight at 4°C [4]. The eluted phage was diluted tenfold in SM buffer to be spotted on the overlay of bacterial lawn to pick a single plaque, and this procedure was repeated seven times to ensure that a single phage type was isolated from the plaques. The isolated phages were amplified in small volumes (5 ml) by mixing TSB of log-phase bacterial culture 10^8^ CFU/ml and 50 µl of isolated phages with titer 10^9^ PFU/ml.

#### Host range assay

The phages’ host range was investigated using the spot assay method against forty-one MDR *A. baumannii* isolates, as previously described [37]. In brief, 10 µL of each phage lysate was added on overlays of bacterial lawns from each isolate and followed by overnight incubation at 37 °C. The phage lysis intensity to the host was assessed based on the clarity of the formed plaques. This assay was carried out in triplicate.

#### Relative efficiency of platting (EOP)

As detailed previously [27]the relative efficacy of plating (EOP) of the phages was determined by counting the number of clear lysis plaques formed after applying 10-fold successive dilutions of the phage onto freshly prepared lawns of each susceptible bacterial isolate. The spotting assay was performed on a double agar overlay, and the plaques were enumerated. The EOP was determined by calculating the ratio of the phage titer on each tested clinical isolate to the titer of host isolate. The EOP ratio was categorized into four levels based on defined quantitative thresholds. Isolates with EOP values ≥ 0.5 were classified as highly efficient. Moderate efficiency was assigned to isolates with 0.1 ≤ EOP < 0.5, while low efficiency corresponded to values in the range of 0.001 ≤ EOP < 0.1. Isolates with EOP values < 0.001 were considered inefficient or resistant.

#### Morphological characterization

The phages were serially diluted to obtain a plaques per plate, when plating 0.1 ml of the diluted stock. Dilutions were prepared in TSB, for plaque formation, 0.1 ml of bacterial culture was mixed with 3.0 ml of molten soft nutrient agar (0.75% agar), followed by the addition of 0.1 ml of the diluted phage. The mixture was poured onto a tryptic soy agar (TSA; Merck, USA) plate. Plates incubated at 37 °C for 18 h. Plaque formation was observed, with the potential for plaque lysis [38]. Plaque size measurement was measured by image j software.

The structure of phages was analyzed using transmission electron microscopy (TEM) in the Faculty of Science, University of Alexandria, Egypt. Initially, 10 µL of the phage, containing 10^10^ PFU/ml was stained with 2.5% uranyl acetate. Subsequently, the sample was attached to a carbon-coated Cu-grid and incubated for 10 min before being analyzed using TEM. The stained phage was imaged using a TEM (1230 JEOL, Tokyo, Japan). The captured images from a JEOL 1230 TEM were measured using ImageJ software version 1.53n [39].

#### Phage production by bioreactor

Phage production was carried out in the BioFlo 120 bioreactor = (Eppendorf, Germany), as previously described, with minor modification [40],1.6 L of TSB was autoclaved in a vessel (Borosilicate glass, 316 L stainless steel). The temperature was 37 °C, and pH 7 was optimized through two front-mounted fixed-speed pumps connected to NaOH and HCL bottles. A single colony of the *A. baumannii* host isolate was resuspended in 200 ml TSB and incubated at 37 °C for overnight culture. After cultivation, 200 ml of the overnight culture was subcultured.

in the bioreactor vessel. The bacterial culture was cultivated at 37 °C and 100 rpm for 4 h until reaching the early log phase (OD_600_ = 0.1). The multiplicity of infection (MOI) of 0.1 was achieved with 0.8 ml of phage (8 × 10^9^ PFU/ml) to the bioreactor vessel by syringe. The agitation was set to 100 rpm, and the gas flow was 2 barg for 5 h. Aliquots were collected in triplicate for phage enumeration and bacterial OD measurement from sample ports using syringes at each time point.

#### Phage stability assay

Phage stability assays were carried out as previously described, with minor modifications [41]. Phage lysates were serially diluted and spotted on their hosts of bacterial culture to evaluate their pH, temperature, and UV stability. The phages’ titers were measured after incubating 1 h with different pH values (2, 3, 4, 7, 10, 11, and 12). Likewise, the temperature stability was assessed by incubating tubes containing phages separately at temperature points − 80, -20, 4, 37, 50, 60, 70, 80 and 90 °C for 1 h. The phage’s tolerance to UV exposure was assessed at 15, 30, 45, 60, 80, and 90. minutes. The stability of the phage was evaluated based on the reduction in phage titer compared to the initial phage titer in SM buffer. For the UV stability assay, the phages were exposed to UV-C light at a wavelength of 254 nm using the built-in UV lamp of the biosafety cabinet (ESCO, Singapore). The distance from the lamp to the sample surface was approximately 60 cm. The lamp’s intensity at this distance was estimated to be 40µW/cm² [42].

#### Time kill kinetics assay

Kinetics of bacterial killing by the isolated phages was investigated at different phage MOIs (0.01, 0.1, 1, 10, 100) using the previously established method of time-killing curve assay [27]. The bacterial strains AB23 and AB24 were used as bacterial host for the isolated phages. Briefly, bacterial culture at 10^6^ CFU/ml was treated with the phage at different MOIs in a 96-well microtiter plate. The bacterial O.D. at a wavelength of 600 nm was determined at 1 h intervals for a duration of 11 h by using FLUOstar^®^ Omega plate reader, BMG LABTECH, Germany. A control of bacterial culture without phage treatment and a blank containing only the culture media were used.

#### Bacteriophage insensitive mutants (BIM)

The BIM frequency was estimated using the previously described method [43]. Briefly, 1 ml of bacterial culture at a concentration 10^7^ CFU/ml was mixed with the phage to achieve MOI of 100. The bacterial strains AB23 and AB24 were used as hosts for isolated phages to assess phage-resistant mutant bacterial cells. After 24 h of incubation at 37 °C, 100 µl of the mixture (phage with its bacterial host) was serially diluted, spotted on TSA, and incubated at 37 °C for 24 h. Phage-resistant mutant bacterial cells were calculated by dividing viable bacterial counts after phage infection by the initial viable count [44].

### Phage genome sequencing

#### Bacteriophage genomic DNA extraction

The genomic DNA of the phage was extracted using phenol-chloroform–isoamyl alcohol method with a minor modification [45]. The phage genomic DNA was extracted from 10 ml of high-titer (10^10^ PFU/ml) and filter-sterilized in a sterile tube. In brief, 1 ml of lysis buffer was added to the phage and then incubated at 56 °C for 1 h. After incubation, 10 ml of phenol/chloroform isoamyl alcohol was added and then centrifuged at 18,000 × g for 10 min to separate the organic phase and aqueous phase; the DNA was collected from the top of the aqueous phase while the organic phase, which contains proteins was discarded. The DNA from the aqueous phase was precipitated overnight at -20 °C by adding a 1:10 volume ratio of 3 M sodium acetate (pH 5.2) and a 2:1 volume ratio of ice-cold isopropanol. By centrifugation at 18,000 × g for 10 min, the precipitated DNA was formed in a pellet. The supernatant was removed, and the pellet was re-suspended in 90% ice-cold ethanol before being transferred to a new 1.5 mL tube. The DNA pellet was washed twice with 70% ethanol, allowed to dry, and then resuspended in 100 ml of nuclease-free water. DNA concentration and quality were evaluated using the FLUOstar Omega Microplate reader (BMG LABTECH, Germany).

#### Genome sequencing and bioinformatics analysis

The Illumina MiSeq platform was used to perform nucleotide sequencing. Library preparation was carried out using the Illumina Nextera tagmentation protocol (Illumina, Cambridge, UK). Quality control of the sequence reads was performed with FastQC (v0.11.9), and de novo assembly was carried out using Unicycle (v0.4.8) via the BV-BRC portal [46]. Genomic visualization, comparison, and orientation were performed using ProgressiveMauve and Ugene software, v43.0 [47, 48]. The single assembled contig was then annotated with the Rapid Annotation using Subsystem Technology Toolkit (RASTtk) pipeline [49]. The annotation process was customized, beginning with the ‘annotate-proteins-phage’ step, followed by ‘annotate-proteins kmer-v2 [50]. Following the RASTtk annotation, a second round of annotation was performed to confirm the assigned functions and to assign functions to proteins that were previously unassigned, using NCBI BLASTp, HHPred, and InterProScan. ARAGORN v1.2.41 was used to identify tRNA genes [51]. A phage genomic map was generated using the Proksee web-based tool [52] by CGView family tools [53].

The phage lifestyle prediction, virulent factor and antimicrobial resistance gene detection, and anti-CRISPR protein were analyzed by PhageScope [54]. In addition, the therapeutic potential of the phage was assessed using PhageLeads [55]which analyzes the phage genome for temperate genetic markers, antimicrobial resistance (AMR), and virulence factors. The transmembrane domains of the phage-predicted proteins were examined using DeepTMHMM, a tool that employs a deep learning algorithm based on a protein language model [56]. The genomic sequence was analyzed with the Phage Depolymerase Finder (PhageDPO, Galaxy Version 0.1.0) prediction tool to identify genes potentially encoding depolymerase functions [57].

#### Phylogenetic analysis

Genome-genome distancing was assessed by ViPtree (proteome-based) [58] and VIRIDIC (nucleotide-based) [59]. VIRIDIC assigned taxa using ICTV species and genus thresholds based on average nucleotide identity (ANI) at 95% and 70%, respectively. Additionally, a phylogenetic tree was constructed on NGPhylogeny.fr [60]using MUSCLE for amino acid sequence alignment [61] and PhyML 3.3.2 with the LG model for amino acid substitution [62]and a BioNJ initial tree to infer the evolutionary relationships of the terminase large subunit protein (encoded by a core gene). A gamma distribution with 4 rate categories was used to handle different mutation rates across sites, and SH-like branch supports were calculated to assess the reliability of the tree branches [63].

### Cell cytotoxicity assay

The phage with a broader host range and better killing capacity was further evaluated for its safety on the human alveolar basal epithelial cell line A549 (ATCC Cat No. CCL-185) by using an MTT assay [27]. A549 cells were cultured in 96-well plates with a seeding density of 10^4^ cells/well, in RPMI-1640 medium supplemented with 10% fetal bovine serum 100 I.U./ml penicillin, and 100 µg/ml streptomycin and incubated for 24 h at 37 °C/ 5% CO_2_. Then, serially diluted phage lysate was added to the cells with different concentrations (10^9^, 10^8^, 10^7^, and 10^6^) and incubated for another 24 h at 37 °C/ 5% CO_2_. After incubation, the medium was removed, and 100 µl of MTT solution (10 µl MTT and 90 µl RPMI) was added and incubated for 4 h at 37 °C and 5% CO_2_. 100 µl of DMSO as a solvent was added and incubated for 15 min in the dark, following OD measurement at 590 nm by using FLUOstar^®^ Omega plate reader, BMG LABTECH, Germany, and the images was taken by an inverted microscope camera (Optika IM-3, Italy).

### Dynamic of phage-bacteria interaction with A549 cells

To assess the effectiveness of the phage in the cell culture line, the bacteria were co-cultured with A549 cell lines and exposed to phage with different MOIs [27]. Bacterial and phage titer were assessed throughout the experiment at different time points, A549 cell viability was assessed only at the end of the assay time (6 h). In brief, human alveolar basal epithelial cell line A549 was used as a model for the in vitro study of phage-bacteria interaction. The cells were cultured in RPMI-1640 medium supplemented with 10% fetal bovine serum, 100 I.U./ml penicillin and 100 (µg/ml) streptomycin at 37 °C/ 5% CO_2_. When it reached confluency, the cells were trypsinzed and cultured in 96-well plates with seeding density 5 × 10^4^ cells/well, incubated at 37 °C/ 5% CO_2_ for 24 h.

At the same time, an overnight culture of AB24 was prepared and incubated at 37 °C. After 24 h, the bacterial culture was centrifuged at 8000 × g for 1 min, resuspended in phosphate buffer saline (PBS) (MP Biomedicals, LLC, Ohio), and OD was measured to obtain a final concentration of 10^5^ CFU/ml. Then, the bacterial culture was centrifuged again and resuspended into complete RPMI without antibiotics.

After checking the cell adhesion on a 96-well plate, the old medium was removed and washed twice with PBS, and 100 µl/well of bacteria suspension in RPMI was added to reach a final concentration of 10^5^ CFU/well. The cells were incubated for 1 h at 37 °C/ 5% CO_2_. Then, the planktonic bacteria were removed, and 100 µl of phage was added with several MOI (0.1, 1, 10, and 100); then, 100 µL of RPMI without antibiotics was added to all the wells. these wells were compared to the control with bacteria and the free cells without bacteria.

The plate count technique was used to enumerate the viable bacterial count and phage titer at (zero, 2, and 6 h). After 6 h, the cells were counted by removing the supernatant, washed twice with PBS, and 40 µl of trypsin was added and incubated for 15 min. Then, 60 µl of complete RPMI was added and mixed well, and cells were counted with trypan blue under a hemocytometer.

### Statistical analysis

Each experiment was carried out in triplicate, with results expressed as the mean ± standard deviation (SD). GraphPad Prism 9.5.1 software was used for data analysis, including generating graphs and conducting one-way ANOVA.

## Results

### Phage isolation and purification

A total of 78% of the *A. baummanii* isolates used for phage isolation were carbapenem-resistant.

The ΦZC2 and ΦZC3 were isolated from hospital wastewater samples using MDR *A. baumannii* AB23 and AB24 as a host bacterium respectively. Phage purification and amplification obtained a pure, high-titer (10^10^ PFU/ml) stock of both phages.

### Phage host range and efficiency of plating (EOP)

**Fig. 1:**
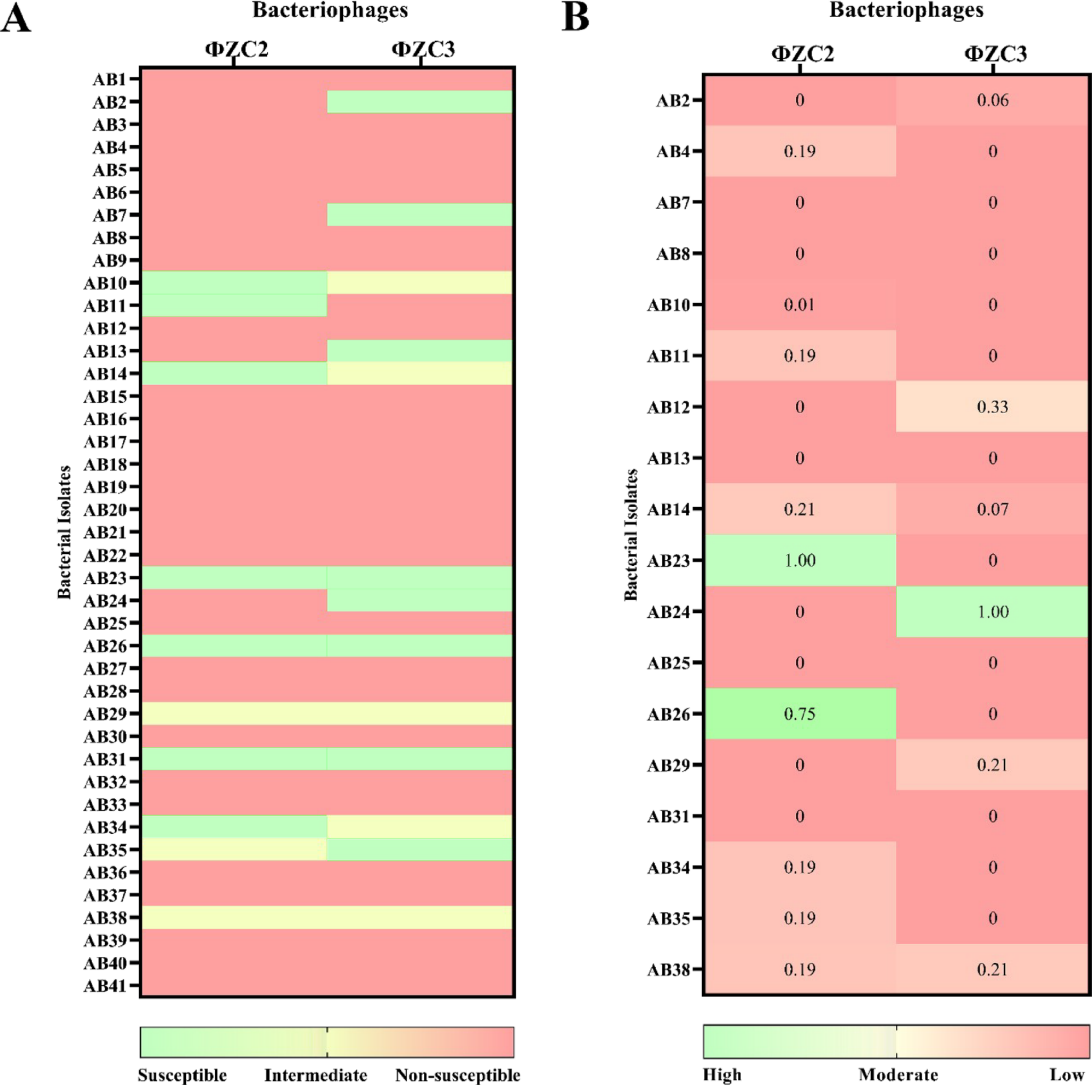
The bacterial isolates’ susceptibility to phages ΦZC2 and ΦZC3. (**A**) The host range of phages ΦZC2 and ΦZC3. Light green color indicates susceptible isolates, light yellow color indicates isolate with intermediate, and light-red color indicates isolate with non-susceptible. (**B**) The efficiency of plating (EOP) of phages ΦZC2 and ΦZC3. Isolates that tested positive in the spot test were selected for the EOP assay. The EOP values were calculated by dividing the phage titer on the test bacterium to that on the host bacterium. Phage production efficiency is categorized as high when EOP ≥ 0.5, moderate when 0.5 > EOP ≥ 0.1, low when 0.1 > EOP > 0.001, and inefficient when EOP ≤ 0.001

The host range of MDR *A. baumannii* phages ΦZC2 and ΦZC3 was determined by spot assays against 41 clinically isolated MDR *A. baumannii*. The results showed that phages ΦZC2 and ΦZC3 have the capacity to lyse 24.3% (*n* = 10) of isolates and 31.7% (*n* = 13) of isolates respectively (Fig. 1A).

Phage ΦZC2 exhibited high EOP values (EOP = 1- 0.75) on both AB23 and AB26 isolates. In addition, both AB34 and AB35 isolates exhibited a moderate EOP value of 0.19. Moreover, AB10, AB24, and AB31 isolates exhibited the lowest EOP value (EOP = 0.01, 0 and 0, respectively). For phage ΦZC3, it was clear that the phage exhibited high EOP values on the AB24 isolate (EOP = 1) and a moderate EOP value on AB12, AB29, and AB38 isolates (EOP = 0.33, 0.21, and 0.21, respectively). For the AB10, AB14, AB23, AB26, AB31, AB34, and AB35 isolates, the EOP value (EOP = 0, 0.07,0,0, 0, 0, and 0), respectively (Fig. 1B) was quite low compared to the other isolates.

### Morphological characterization

The plaque morphology of phages ΦZC2 and ΦZC3 was observed. On the *A. baumannii* lawn, it was found that the plaques were surrounded by haloes with a diameter of 8.168 mm and 7.888 mm, respectively, which could indicate the presence of depolymerase activity. The diameters of the plaques without the surrounding halo zones were 4.3133 mm for ΦZC2 and 1.7981 mm for ΦZC3.

**Fig. 2:**
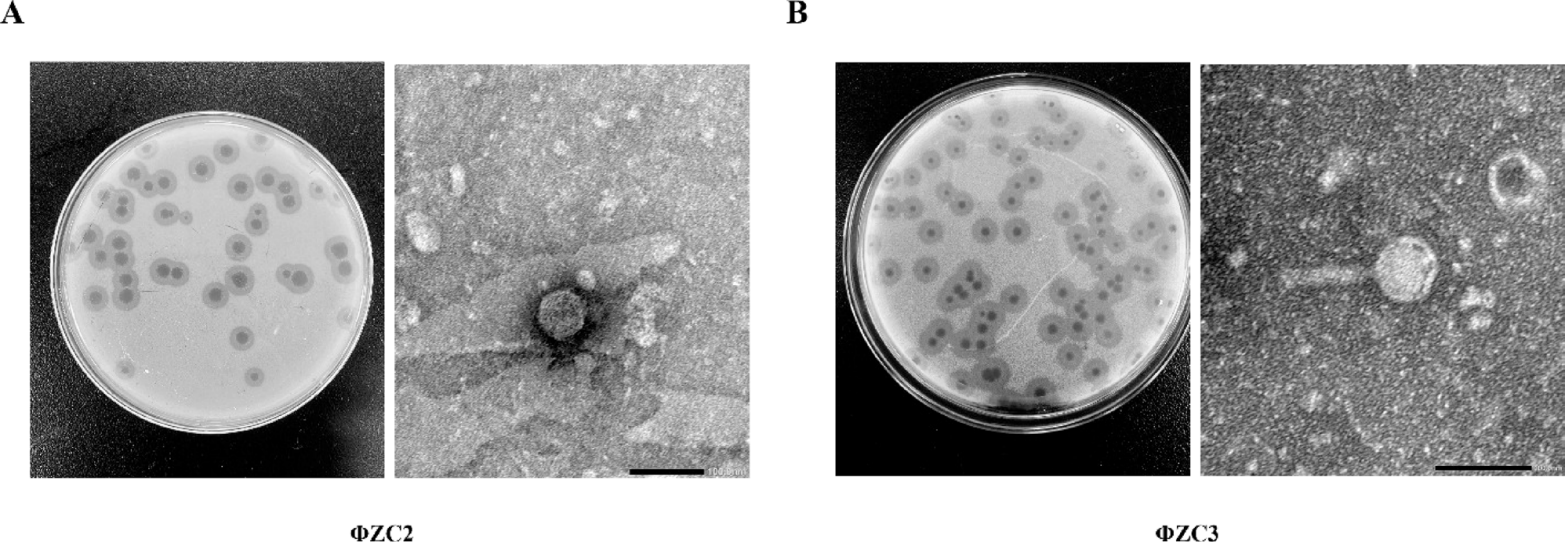
Morphological characterization. (**A**) Morphology of phage plaque and TEM micrographs for ΦZC2 (**B**) Morphology of phage plaque and TEM micrographs for ΦZC3

Moreover, the morphology of phage particles was observed under a transmission electron microscope (TEM), and it was observed that the ΦZC2 belongs to podovirus as it consists of an icosahedral head with a diameter of 60.483 ± 1.26 nm and a very short tail with a length of 32.009 ± 1.42 nm. Similarly, phage ΦZC3 belongs to myovirus, and contains an icosahedral head with a diameter of 66.807 ± 3.05 nm × 62.592 ± 1.77 nm, and a long tail with a length of 90.92 ± 1.72 nm (Fig. 2A and B).

### Phage production using bioreactor

**Fig. 3:**
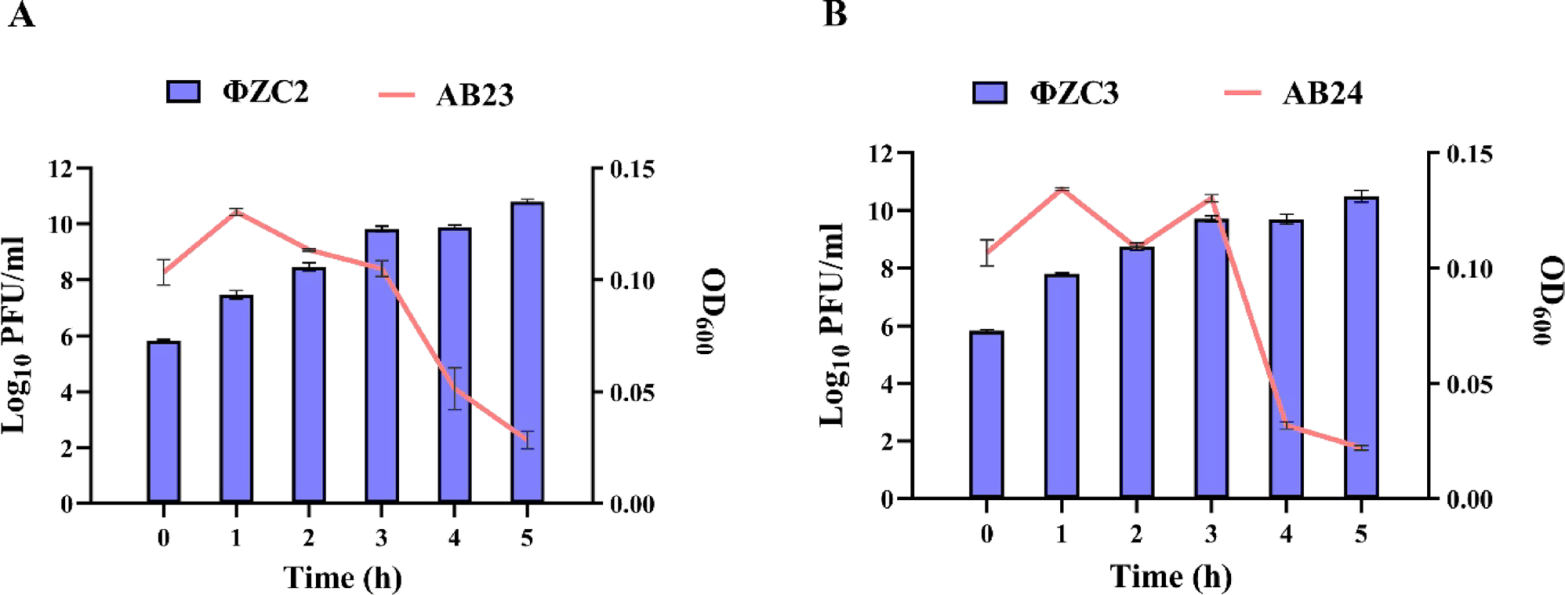
Phage production using a bioreactor. Phage ΦZC2 targeting bacterial culture AB23 and phage ΦZC3 targeting bacterial culture AB24 are represented in panels A and B, respectively. Each bacterial culture had a total volume of 2 L in TSB, adjusted to 10^6^ CFU/ml and infected with the corresponding phage at a multiplicity of infection (MOI) of 0.1. The blue bars represent the phage titer (Log₁₀ PFU/ml) measured at various time points, while the red line indicates the optical density (OD₆₀₀) of the respective bacterial cultures. Error bars represent the standard deviation from triplicate technical measurements

The results showed that the titer of phage ΦZC2 and ΦZC3 reached approximately 10^10^ PFU/ml in 2 L. In brief, ΦZC2 increased until it reached 10.09 ± 0.1233 log_10_ PFU/ml, and the OD600 measurements indicated that the bacterial concentration of AB23 decreased sharply after 3 h to reach 0.0283 ± 0.00404 (Fig. 3A). Likewise, phage titer of phage ΦZC3 was increased to 10.4924 ± 0.19941 log_10_ PFU/ml, and the OD600 measurements indicated that the bacterial concentration of AB24 decreased sharply after 3 h to reach 0.03167 ± 0.00153 (Fig. 3B).

### Phage pH, temperature, and UV stability

The stability of phages (ΦZC2 and ΦZC3) was examined against different environmental conditions, including pH, Temperature, and UV.

**Fig. 4:**
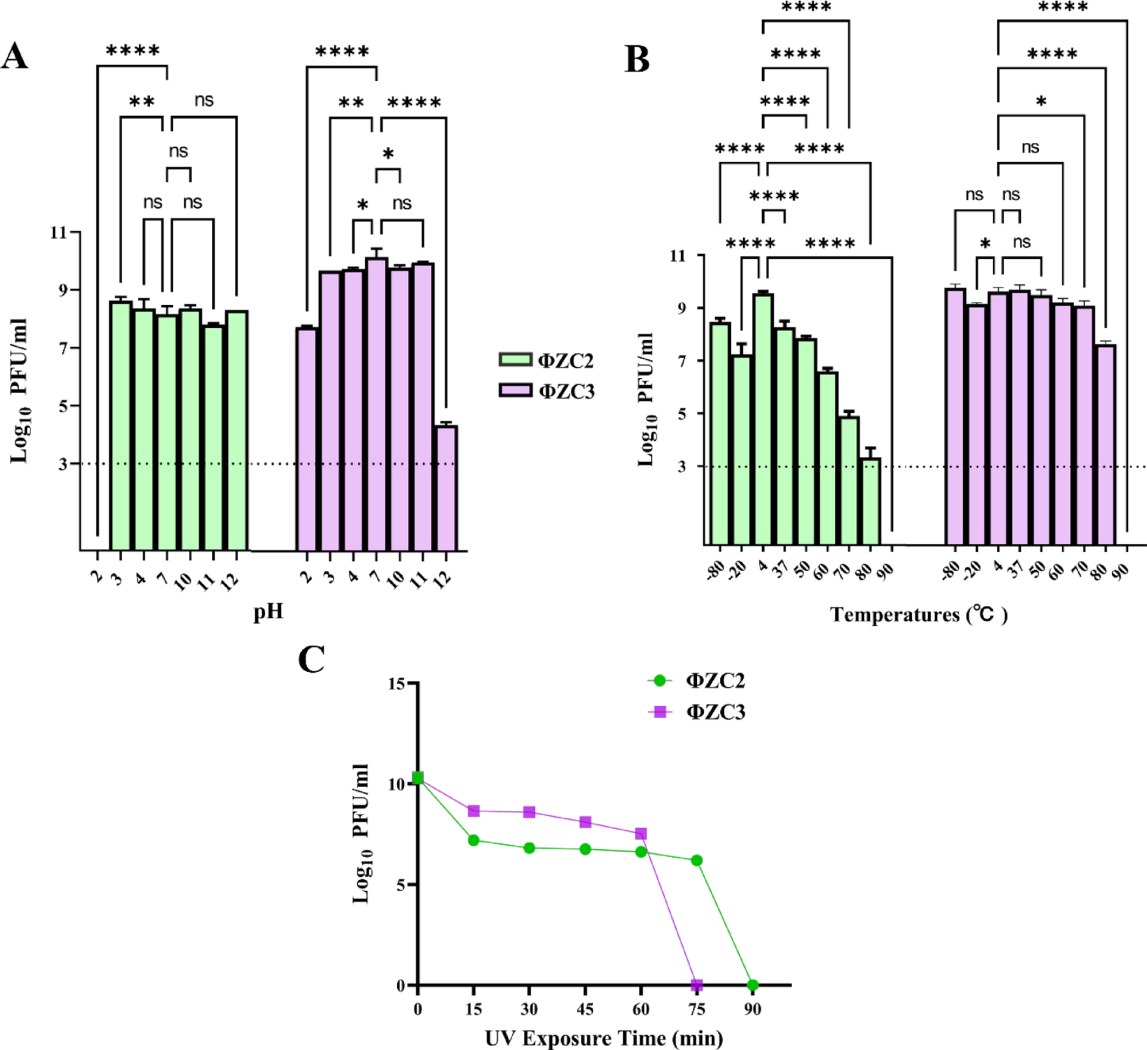
Characterization for the two lytic phages. (**A**) pH Stability, (**B**) Temperature Stability, and (**C**) UV Stability for ΦZC2 and ΦZC3, respectively

Phage ΦZC2 demonstrated pH at 3, 4, 10, 11, and 12 with approximately 10^8^ PFU/ml compared to pH 7. However, it was sharply reduced under the limit of detection at higher acidity (≤ pH 2.0). Phage ΦZC3 also demonstrated stability at pH 3, 10, and 11 with approximately 10^9^ PFU/ml compared to pH 7. However, it was reduced by approximately 2 logs at the acidity (≤ pH 2.0) and 5 logs at alkalinity (≥ pH 12.0) conditions (Fig. 4A).

Phage ΦZC2 titer was approximately 10^9^ PFU/ml at 4 °C. it was stable at -80, 37, and 50 °C with approximately 10^8^ PFU/ml. However, the titer at -20, 37, 50, 60, 70, 80 °C was decreased by 2, 1, 1, 2, 4, 6 logs, respectively, compared to 4 °C. On the other hand, phage ΦZC3 titer was approximately 10^9^ PFU/ml at 4 °C. it was stable at -80, 37, 50, 60, and 70 °C with approximately 10^9^ PFU/ml. However, the titer at -20 and 80 was decreased 1 and 2 logs respectively compared to 4 °C. Both phages became under the limit of detection at 90 °C (Fig. 4B).

In addition, the phage ΦZC2 and ΦZC3 exhibited high UV stability activities and showed complete inactivation by 75 and 90 min, respectively (Fig. 4C).

### Time-kill kinetics assay

**Fig. 5:**
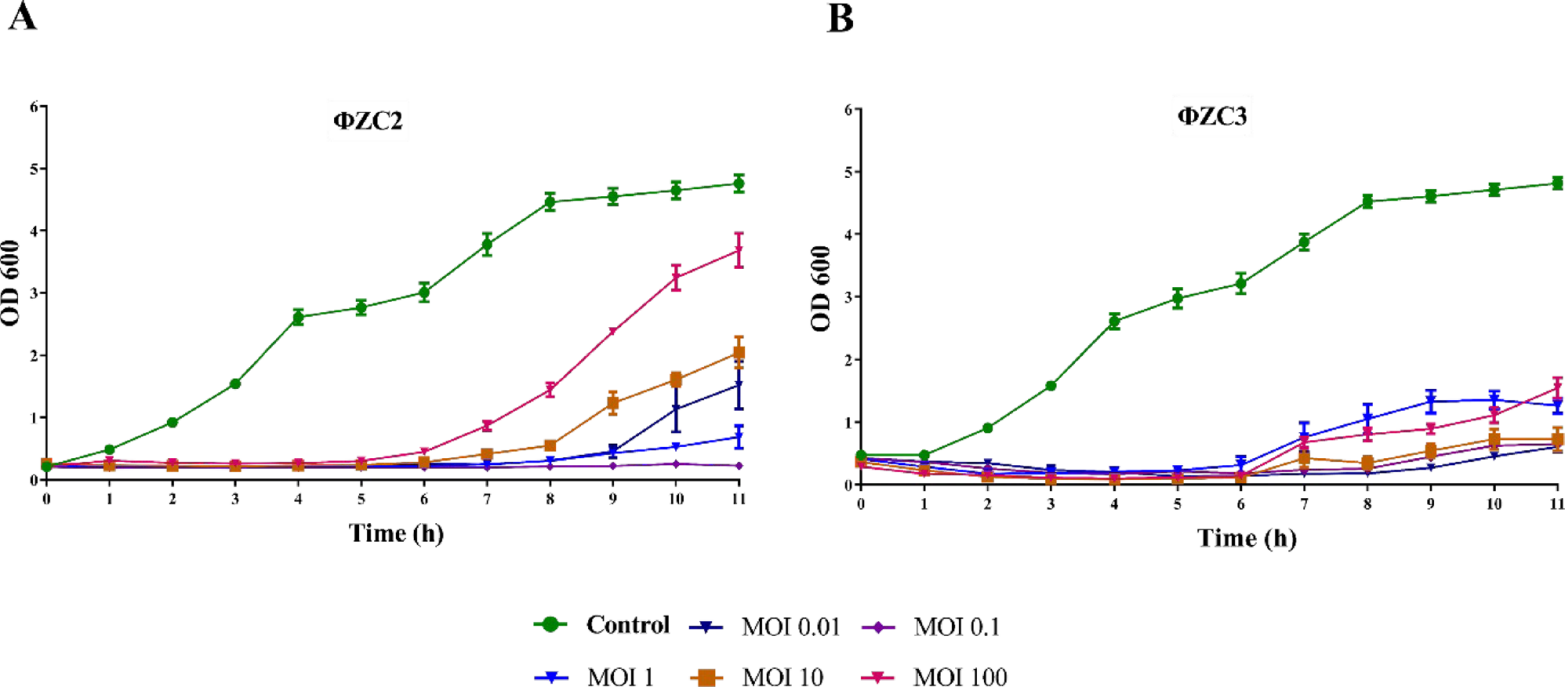
Time-kill kinetics assay of MDR *A. baumannii* isolates. (**A**) AB23 strain treated with ΦZC2 and (**B**) AB24 isolate treated with ΦZC3 at different MOIs (0.01, 0.1, 1, 10, and 100) for 11 h incubation at 37 °C with shaking at 100 RPM

Phage ΦZC3 exhibited a significant reduction in optical density (O.D.) of AB23 bacterial culture at 600 nm at MOIs of 100 and 10 after 11 h, in contrast to Phage ΦZC2, indicating lytic activity. In brief, the groups treated with ΦZC2 phage against AB23 showed a reduction in the O.D. at 600 nm, in contrast to the control (AB23 isolate). The O.D. of bacteria treated with MOIs 0.01, 0.1, 1, and 10 was reduced rapidly. The control had an O.D. of 4.7 ± 0.2 after 11 h. At a MOI of 0.01, the O.D. was 1.522 ± 0.65, at MOI of 0.1, the O.D. was 0.226 ± 0.038, at MOI of 1, the O.D. was 0.688 ± 0.25, at MOI of 10, the O.D. was 2.041 ± 0.494, and at MOI 100, the O.D. was 3.681 ± 0.549. Bacteria began to show an increase in the O.D. readings after 6 h of infection with MOIs 1, 10, and 100, which may indicate the likely presence of phage resistance (Fig. 5A).

ΦZC3 phage displayed a reduction in the O.D. of AB24 bacterial culture at 600 nm. Moreover, the bacteria treated with higher MOIs (10 and 100) presented a quick reduction in the O.D. readings. After 11 h, the O.D. of the control was 4.8 ± 0.1; while it was at 0.677 ± 0.1 MOI 0.001, 0.891 ± 0.424 at MOI 0.1, 1.238 ± 0.328 at MOI 1, 0.732 ± 0.452 at MOI 10 and 1.969 ± 0.257 at MOI 100. At MOIs 0.01, 0.1, 1, 10, and 100, the O.D. of the bacterial culture was increased after 6 h, which might reflect the probable phage resistance (Fig. 5B).

### Bacteriophage insensitive mutants

The BIM frequency, which indicates the proportion of bacteriophage-insensitive mutants, is higher for ΦZC3 (1.83 × 10^− 2^ ±1.26 × 10^− 2^) compared to ΦZC2 (1.67 × 10^− 2^ ±5.77 × 10^− 3^). This suggests that ΦZC3 has a slightly higher occurrence of phage-resistant mutants.

### Genomic characterization of ΦZC2 and ΦZC23

#### Analysis and annotation of genomes

**Fig. 6:**
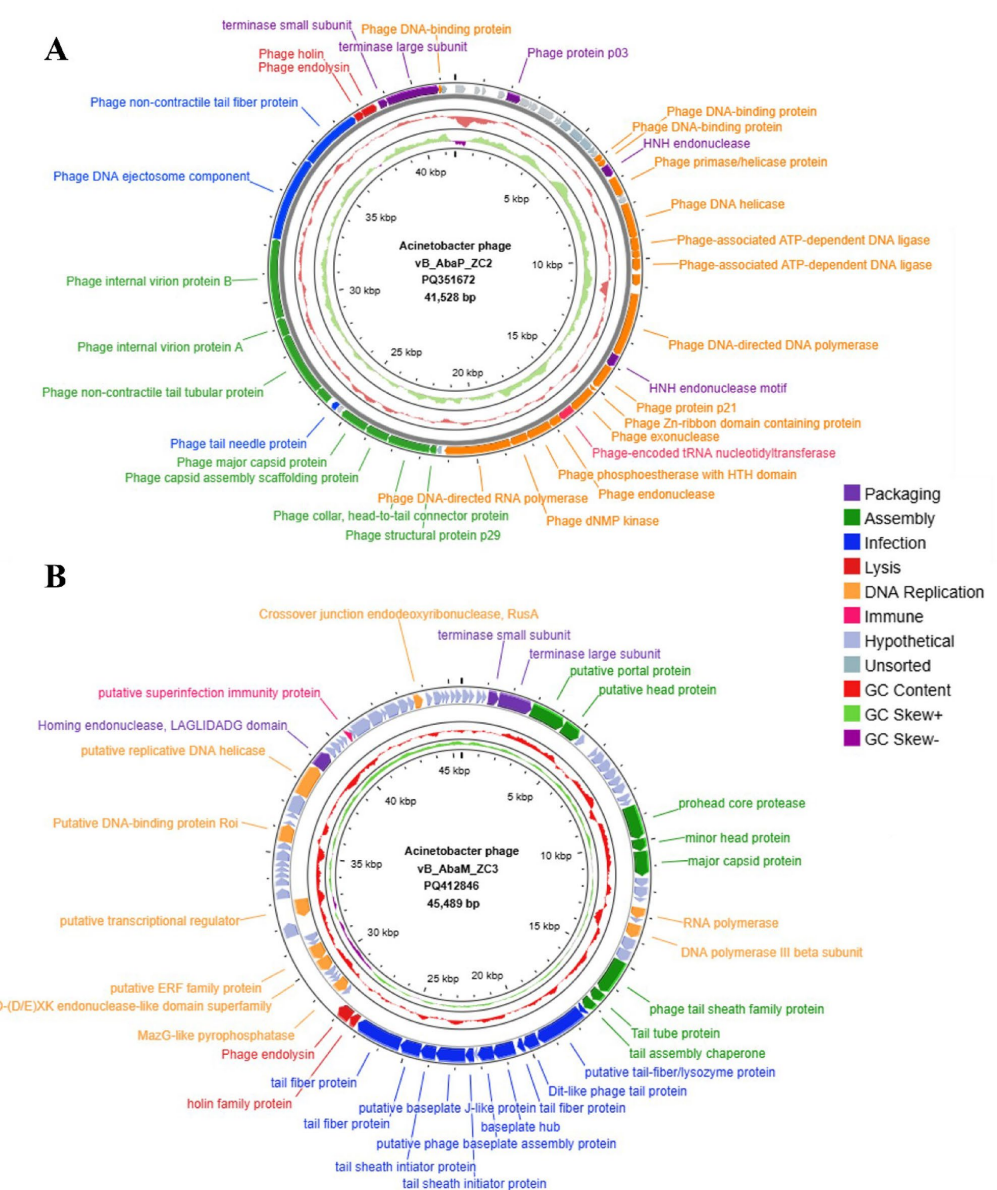
Genomic map of ΦZC2 and ΦZC3. The color coding indicates the coding sequences (CDS), grouped by the predicted functional categories: packaging (purple), assembly (green), infection (blue, lysis (red), DNA replication (orange), immune (pink), phage and hypothetical proteins (shades of grey), GC content (light red), and GC skew (light green and purple). The genomics maps were created on Proksee server

The genomes of the phages were sequenced, assembled, annotated, and submitted to GenBank as *Acinetobacter* phages. ΦZC2 has a double-stranded DNA genome measuring 41,528 bp (accession PQ351672), and ΦZC3 also has a double-stranded DNA genome with a length of 45,489 bp (accession PQ412846). Both ΦZC2 and ΦZC3 phages are tailed and are classified within the *Caudoviricetes* class. The ΦZC2 phage is a member of the family *Autographiviridae*, sub-family *Beijerinckvirinae*, and genus *Friunavirus*, while the ΦZC3 phage belongs to the genus *Obolenskvirus* [64]. The open reading frames (ORFs) predicted in the complete genome of ΦZC2 (Fig. 6A) and ΦZC3 (Fig. 6B) phages were 55 and 88 respectively, including function categories which are DNA genome packaging, structure assembly protein, infection-associated proteins, bacterial cell lysis proteins, DNA replication proteins, and immune proteins. Each phage had 33 ORFs assigned to various protein functions.

**Fig. 7:**
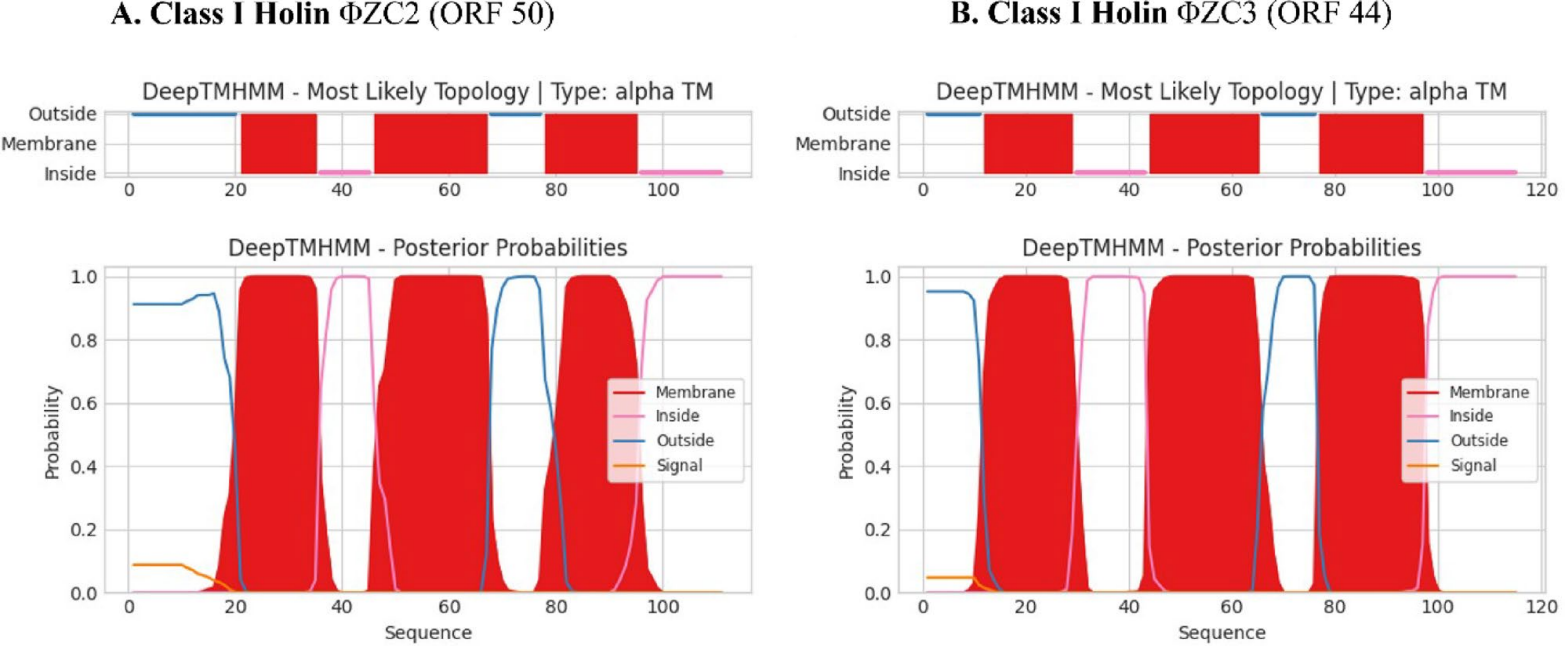
Predicted topology of putative holins encoded by ORFs 50 and 44. DeepTMHMM was used to predict the topology of ORF 50 (**A**) and ORF 44 (**B**), respectively. The top part of each chart represents the topology of predicted domains in correspondence to the amino acid sequence: transmembrane (in red), intracellular (in pink), and extracellular (in blue). The probability of predicted topology is presented in the bottom part

No tRNA gene was found for either of the phages. The phages lifestyle prediction was virulent. There were no antimicrobial resistance genes and anti-CRISPR proteins detected. PhageLeads did not identify any genes associated with a temperate lifestyle, nor were any antimicrobial resistance or virulence genes detected. DeepTMHMM predicted class I transmembrane topology, which led to identifying the ΦZC2 phage, a gene ORF 50 encoding holin (Fig. 7A). Nevertheless, a ΦZC3 phage was found where DeepTMHMM identified a class I transmembrane topology, for holin encoded by ORF 44 (Fig. 7B). The PhageDPO tool detected depolymerase activity in the ΦZC2 phage, non-contractile tail tubular protein (ORF 45) with 98% prediction. However, in the ΦZC3, putative tail-fiber/lysozyme protein (ORF 33) with 88%.

#### Phylogenetic tree

**Fig. 8:**
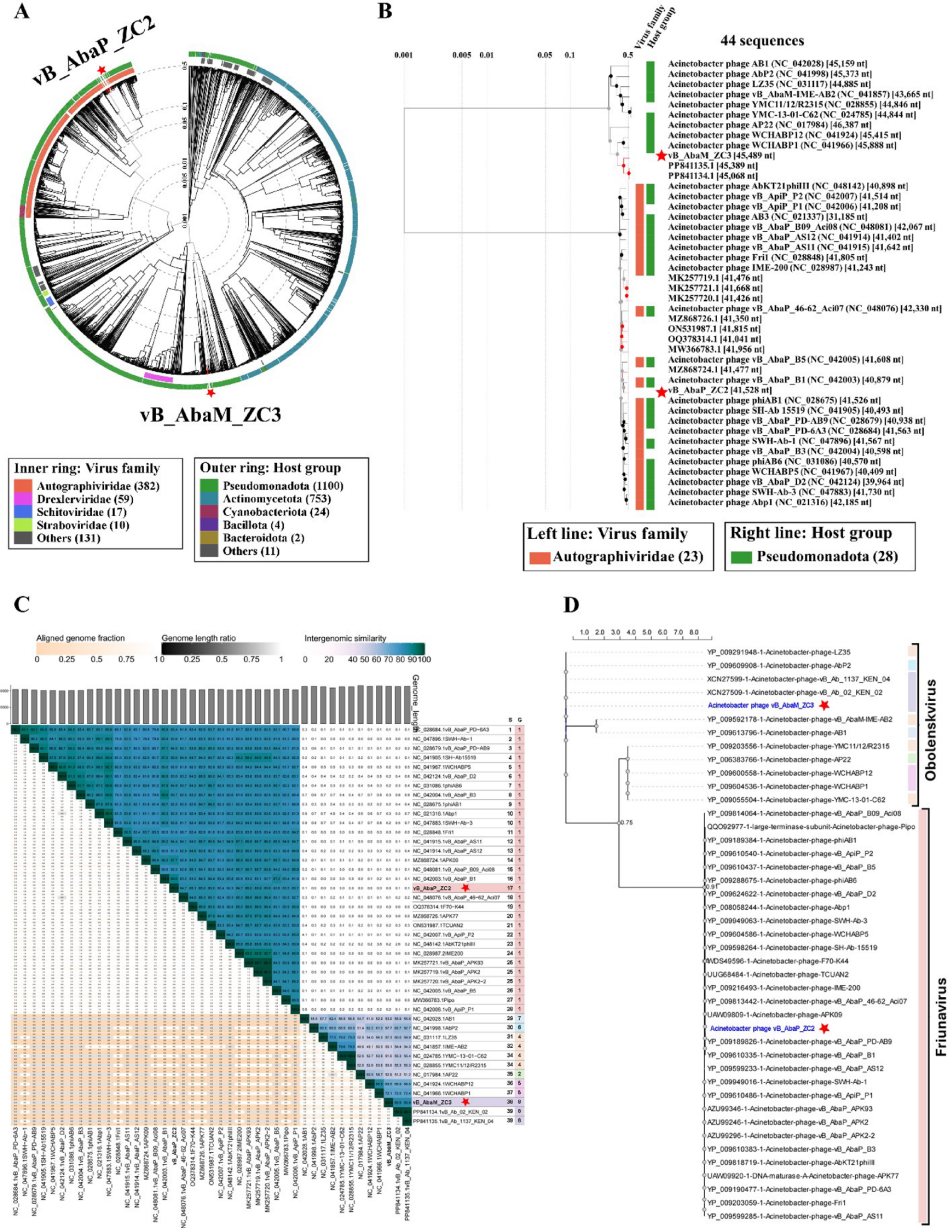
Phylogenetic analysis of phages vB_AbaP_ZC2 and vB_AbaM_ZC3 (indicated by red stars). (**A**) The circular proteomic tree produced by ViPtree represents the proteomic relationships of Acinetobacter phages with the isolated phages vB_AbaP_ZC2 and vB_AbaM_ZC3. (**B**) Rectangular proteomic tree highlighting closely related phages with high ViPtree similarity scores (SG > 0.5). (**C**) Heatmap representing intergenomic similarities between 41 Acinetobacter phages generated using the VIRIDIC tool. The aligned genome fraction, genome length ratio, and intergenomic similarity are displayed for each pairwise comparison. The color gradient from light to dark teal represents increasing intergenomic similarity. Phages are grouped based on genomic similarities, with ‘S’ referring to isolate-level clustering and ‘G’ referring to genus-level clustering. Species and genus classification are based on ICTV thresholds for ANI. (**D**) Maximum likelihood phylogenetic tree constructed using the NGPhylogeny.fr tool with MUSCLE for amino acid sequence alignment and PhyML 3.3.2. SH-like branch supports are provided

Phylogenetic analysis of the newly isolated *Acinetobacter* phages vB_AbaP_ZC2 and vB_AbaM_ZC3 was performed using multiple tools to assess their genomic and proteomic relationships. A circular proteomic illustrated the proteomic similarities among the two *Acinetobacter* phages vB_AbaP_ZC2 and vB_AbaM_ZC3 (Fig. 8A). A rectangular tree further highlighted phages with high proteomic similarity (Fig. 8B). A heatmap displayed intergenomic similarities among 41 *Acinetobacter* phages (Fig. 8C). Lastly, a maximum likelihood phylogenetic tree was constructed to compare the terminase large subunit protein sequences of vB_AbaP_ZC2 and vB_AbaM_ZC3 with homologs from related phages in the genera *Obolenskvirus* and *Friunavirus*, based on NCBI taxonomy. (Fig. 8D). This comprehensive analysis provides a detailed understanding of the phylogenetic relationships of these two newly isolated phages.

### Cytotoxicity assay

**Fig. 9:**
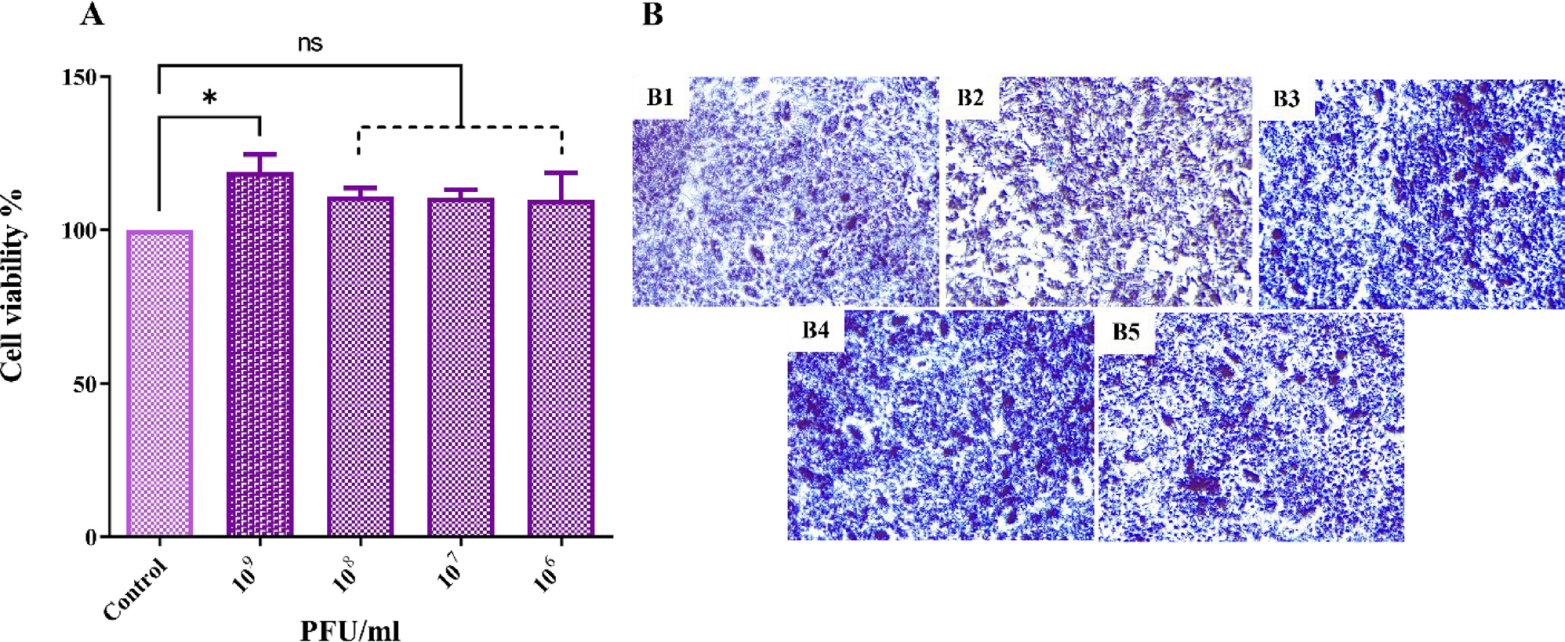
The cell viability of ΦZC3 and cell images under fluorescence microscope on A549 cells. (**A**) different concentrations of ΦZC3 (10^9^ to 10^6^ PFU/ml) compared to control. (**B**) cell images, control (B1), 10^9^ (B2), (B3)10^8^, (B4) 10^7^, and (B5) 10^6^ PFU/ml

The efficacy and safety of phage treatment and the cytotoxicity of ΦZC3 phage were tested against A549 alveolar cell lines. Phage ΦZC3 has no cytotoxicity effect on A549 cells, with a viability above 100% at all tested phage concentrations, as the cell viability did not decrease with different titers (10^9^ to 10^6^ PFU/ml) compared to the control of A549 cells only (Fig. 9A). The cells images with different titers compared to the control indicated the safety of phage therapy on epithelial cell lines (Fig. 9B).

### Dynamic of ΦZC3 phage-AB24 bacteria interaction with A549 cells

**Fig. 10:**
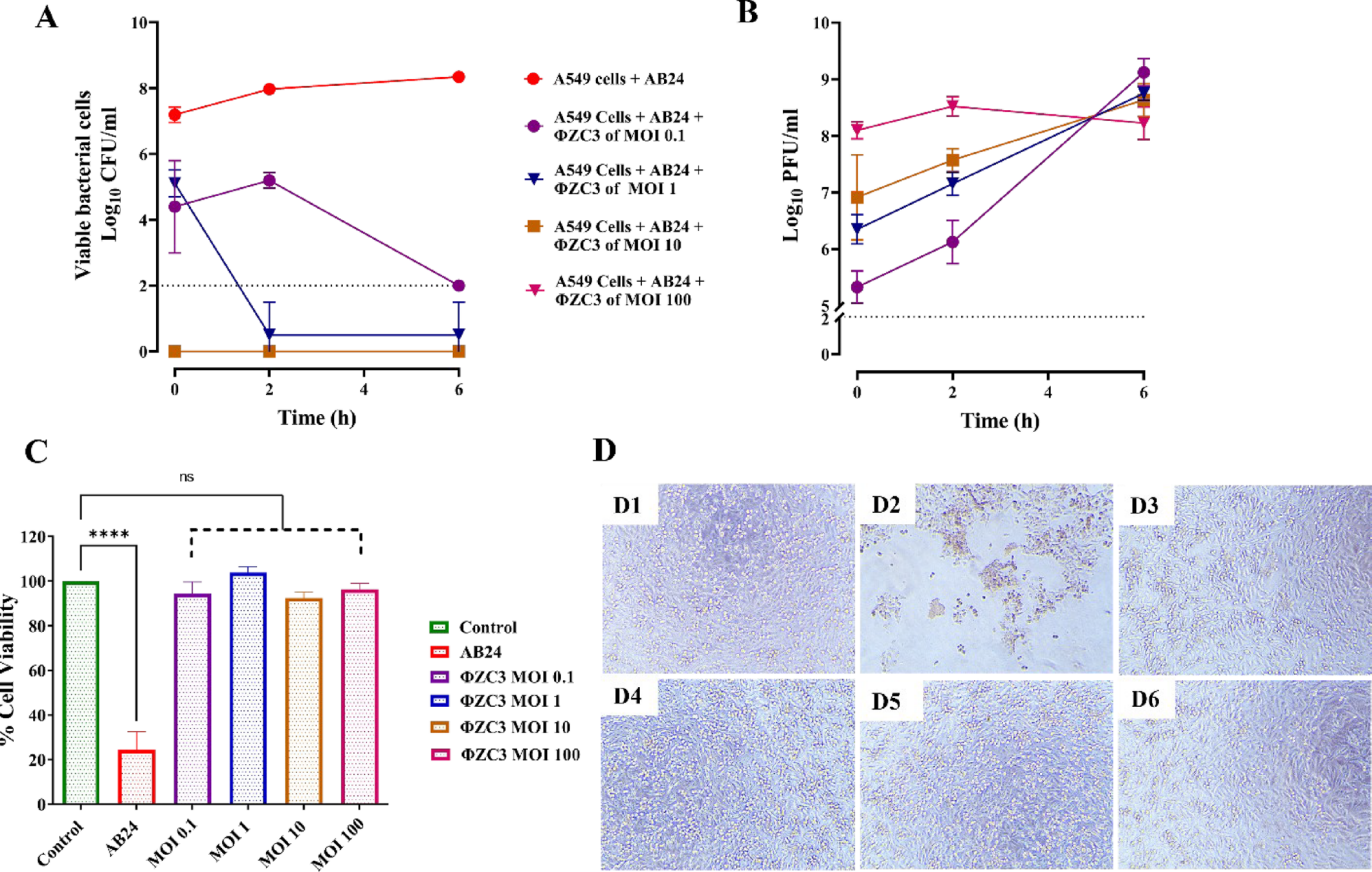
Dynamics of ΦZC3 phage and AB24 bacteria interaction with A549 Cells. Viable bacterial cells (**A**), phage titer (**B**), cell viability % (**C**) and A549 cell images (**D**) where (D1) is the control, (D2) cells with bacteria (D3), cells after phage treatment (MOI:100), (D4) cells after phage treatment (MOI:10), (D5) cells after phage treatment (MOI:1), and (D6) cells after phage treatment (MOI:0.1)

At zero time of phage infection to the cells, the bacterial concentration decreased sharply by 2.5 log at MOIs 0.1 to 1 for ΦZC3 phage and decreased by 5 logs at MOIs 10 and 100, compared to the control. The number of bacterial cells significantly reduced for all MOIs at 2 h and 6 h (approximately 10^3^ CFU/ml; *P* < 0.05) (Fig. 10A).

In addition to the CFU reduction, phage ΦZC3 titer was increased throughout the time points, indicating successful phage production (Fig. 10B).

For the A549 cell line, the cell viability was assessed after 6 h, and the results showed that with all selected MOIs, the cell numbers were unaffected compared to the control (bacterial cell only) (Fig. 10C and D).

## Discussion

Multidrug-resistant (MDR) *Acinetobacter* species have been dramatically increasing.

over the past decade and showing a significant healthcare concern globally to control MDR infection [65]bacteriophage is considered a promising alternative when selectively isolated on their bacterial hosts [25, 33]. During This study two phages (ΦZC2 and ΦZC3) were successfully isolated from a hospital wastewater sample. The analysis of phage host range and EOP demonstrated that phage ΦZC2 could infect 24.3% of the *A. baumannii* isolates, while ΦZC3 phage could infect about 31.7% of the 41 *A. baumannii* isolates. However, the host ranges of phages like Abp4 could infect 13.6% of the 22 *A. baumannii* isolates [66]. These results suggest the potential for using isolated phages in clinical studies, particularly when combined with a phage cocktail in future studies.

Both phages exhibited stability over a range of pH, temperatures, and UV exposure for approximately 1 h. These results were supported by previous research [41, 67, 68]as well as by investigations using several bacterial species [69, 70]. The time-killing curve results showed a rapid decline in bacterial growth following treatment with ΦZC2 and ΦZC3 phages separately, each targeting its specific bacterial host (AB23 and AB24), indicating the strong lytic potential of the phages. Throughout the first 5 h, a significant reduction was observed with all MOIs; however, following 8 h of infection, bacterial regrowth was observed, likely due to the emergence of resistant bacteria because of resistance mechanisms developing after antimicrobial suppression [71]. Although the development of the resistant bacteria, MOI 0.1, showed the best infection concentration for the two phages compared to other MOIs. At the same time, the result indicated that phage ΦZC3 could control the bacterial growth for a long time (11 h) at OD > 2 with all MOIs (0.01, 0.1, 1, 10, 100). The phage ΦZC3 result highlights the phage’s potential as a therapeutic agent for a long time, which is why it was selected for further studies on A549 lung epithelial cells.

The sequencing and annotation of the genomes of *Acinetobacter* phages ΦZC2 and ΦZC3 provide valuable insights into their genomic structure and potential functionalities. ΦZC2 features a double-stranded DNA genome of 41,528 bp, which is consistent with vB_AbaP_B1 reported in a phylogenetic tree study with an average length of 40,879 bp [72]. While ΦZC3 has a slightly larger genome of 45,489 bp, which is consistent with the *Acinetobacter* phage vB_Ab_1137_KEN_04 phage reported in a phylogenetic tree study with an average length of 45,389 [73].

The genomes of phages ΦZC2 and ΦZC3 have no lysogeny markers, virulence factors, or antibiotic resistance genes, assuring their safety for therapeutic applications [74]. The presence of an immune protein, such as the phage-encoded tRNA nucleotidyltransferase and putative superinfection immunity, may enhance its ability to persist in a competitive microbial environment [75, 76].

Both phages produce small plaques on a lawn of MDR *A. baumannii*, and the presence of haloes around these plaques suggests that the phage may produce a depolymerase that can degrade polysaccharides. These results were confirmed with the whole genome analysis, as the PhageDPO program identified depolymerase activity in the ΦZC2 phage’s non-contractile tail tubular protein (ORF 45) with a 98% prediction, and in the ΦZC3, the putative tail-fiber/lysozyme protein (ORF 33) with 88% similarity. The same results were mentioned in other studies, as in a recent study, phage vABPW7 also produced small plaques on a lawn of MDR *A. baumannii*, and the surrounding halo indicates that the phage might produce depolymerase with the ability to break down polysaccharides [27]. Likewise, the “K2/K93, K32, K37, K44, K48, K87, K89, and K116” phages could form clear plaques surrounded by haloes on *A. baumannii* host strains, which indicates the breakdown of polysaccharide capsules by phage structural depolymerase [77].

Prior research has indicated that MDR *A. baumannii* could adhere to, colonize, and invade human epithelial cells, subsequently leading to intracellular proliferation, dispersion to other tissues, persistence, and the activation of cell death pathways [78]. As a result, the A549 cell line, which is a human lung epithelial cell line, is used as a model to examine the pneumonia infection caused by MDR *A. baumannii* infections [79, 80].

Several studies have confirmed that the phages have no harmful effect on human skin fibroblast (HSF) and (HT-29) cells [27, 78, 81]. Similarly, the ΦZC3 exhibited no cytotoxicity against adenocarcinomic human alveolar basal epithelial cells (A549), consistent with a previous study on the A549 cell line that evaluated the phage’s ability to reduce bacterial adhesion and demonstrated a dose-dependent reduction in the total number of adherent MDR *A.* baumannii, with no harmful effects on the cells [21].

In the phage dynamic experiment, the ΦZC3 phage reduced the bacterial count at several time points without affecting the viability of A549 cells. This indicates that the phage could reduce the invasion and formation of intracellular bacteria. Previous studies showed that the phage-bacterial interaction reduces colonization [71]and the presence of phages influences the interaction between bacterial and mammalian hosts^87^. Bacterial virulence is a multifaceted phenomenon and is influenced by several factors. More studies are needed for the detailed molecular mechanism of the virulence factors of *A. baumannii*, and its interaction with the decreased attachment to cell lines [82].

In conclusion, this study highlights the ability of phages ΦZC2 and ΦZC3 in targeting MDR *A.baumannii*. Both lytic phages demonstrated broad-spectrum activity, and their stability under different environmental conditions enhances their potential for practical application. Notably, phage ΦZC3 was identified as a promising therapeutic for pneumonia infection caused by MDR *A.baumannii.* The bacterial reduction effect observed in A549 cells infected with *A.baumannii* without affecting the cell viability suggests that ΦZC3 could be a potential alternative therapy. Further investigations into molecular markers and in vivo studies for understanding the host-pathogen relationship and optimizing phage treatment in clinical applications, particularly for pneumonia and other critical infections.

## Data Availability

Sequence data of the bacteriophages have released in NCBI:ΦZC2: accession PQ351672ΦZC3: accession PQ412846.
